# Pain perception of older adults in nursing home and home care settings: evidence from China

**DOI:** 10.1186/s12877-018-0841-0

**Published:** 2018-07-03

**Authors:** Yuebin Xu, Nan Jiang, Yean Wang, Qiang Zhang, Lin Chen, Shuang Ma

**Affiliations:** 1 School of Social Development and Public Policy, Beijing Normal University, 19 Xinjiekou Wai St, Beijing, 100875 China; 2 Columbia University School of Social Work, 1255 Amsterdam Ave, New York, NY 10027 USA; 3 School of Social Development and Public Policy/ Innovation Center for Risk Resilience, Beijing Normal University, 19 Xinjiekou Wai St, Beijing, 100875 China; 4 Beijing Normal University, Zhuhai, No 18 Jinfeng Road, Tangjiawan, Zhuhai City, Guangdong Province China; 5 School of Management, Beijing University of Chinese Medicine, 11 North Third Ring Road, Beijing, 100029 China

**Keywords:** Pain, Long-term care, Nursing home, China, Older adults

## Abstract

**Background:**

In the past decade, the number of long-term care (LTC) services for older adults in China has grown annually by an average of 10%. Older adults, their family members, and policymakers in China are concerned about patient outcomes in different care settings because older adults who have a similar functional status and LTC needs may choose either nursing home care or home care. The aim of this study was to compare pain perception in nursing home care and home care settings for physically dependent older adults in China.

**Methods:**

Multi-stage sampling method was used to recruit respondents aged 65 and older from Yichang City, China, in 2015. The researchers employed a two-step analytical strategy—zero-inflated ordered probit regression followed by propensity score matching method—to model the effect of contrasting residence types on pain perception.

**Results:**

Zero-inflated ordered probit regression analysis with participants unmatched (*n* = 484) showed that compared with older adults who received home care, those who received nursing home care did not have more severe pain (*β* = 0.088, *SE* = 0.196, *p* = 0.655). After propensity-score matching, the research found that older adults in the home care group perceived less pain compared with the nursing home group (*β* = 0.489, *SE* = 0.169, *p* = 0.004).

**Conclusions:**

The older adults who received home care perceived significantly less pain than the nursing home residents. The pain of older adults may differ based on the type of LTC services and therapy intensity they received, and home care might lead to less pain and better comfort than nursing home care.

## Background

Pain is a well-documented public health problem among older adults in China [1–5]. It is defined as “an unpleasant sensory and emotional experience correlated with actual or potential issue damage or described in terms of such damage” [6]. Previous studies in China have shown approximately 30.9% of the urban older adults and 38.7% of the rural older adults were reported to have pain [7]. Other national representative surveys, such as China Health and Retirement Longitudinal Study, found similar results [8].

In recent years, pain has become an increasingly important measure of well-being and quality of life for older adults [9, 10]. It influences many aspects of an individual’s physical, emotional, and social functioning [11–13]. Evidence indicates that pain has a stronger association with health outcomes, such as depression, anxiety, social isolation, sleep disorders, malnutrition, loss of appetite, cognitive disorders, and delirium [1, 3, 5, 14, 15]. With their high physical vulnerability, pain substantially decreases older people’s activities of daily living (ADLs) and reduces quality of life, which in turn leads to higher health care cost [9, 16, 17]. Therefore, improving pain management, particularly in long-term care system, should be regarded as a priority for healthcare services.

Several individual characteristics appear to influence pain prevalence, including care settings. On the one hand, pain is common in nursing home residents and 45 to 80% of nursing home residents suffer from substantial pain [4, 18]. Kane et al. [19] and Lee et al. [20] found that the majority of nursing homes in the United States and Korea are not adequately staffed to provide appropriate pain management. On the other hand, almost 30% of home care residents in Canada were evaluated as having current daily pain, most commonly associated with arthritis [21]. To date, however, little research has been conducted to examine pain perception among older adults in community and institutional care settings separately in China.

The current study aims to evaluate the pain perception in nursing home care and home care settings for physically dependent older adults in Yichang City, China. Pain is an important outcome measure for care settings given that it is a crucial factor in planning and providing care for older adults [17, 18, 22]. Although the introduction of professional aging care modified the system of care service provision to older adults in China, it was not known whether it improved pain perception of older adults. The future of the long-term care (LTC) system may be uncertain due to some unanticipated challenges in its financing and service evaluation, but understanding whether pain outcomes improved after older adults received LTC is still important. This study contributes to the ongoing policy discussion in searching for efficient service choices to meet the mounting LTC needs of older adults in China by analyzing survey data from nursing home care and home care settings in Yichang City.

### Long-term care service provision in China

With the rapid demographic shifts and former one-child policy, aging population poses serious challenges for the health and long-term care systems in China. Family care at home is rooted in the Chinese principle of filial piety and is still the predominant form of care provision today [23]. However, due to the growing number of smaller families with fewer children as well as the rise of population mobility, the availability of adult children to provide support and care for older parents is declining. In urban areas, the emerging “4–2-1” family structure (four grandparents, two parents, and one child) is a potential risk factor for the well-being of families. In rural areas, rural-urban migration in recent more than two decades has made older adults geographically distant from their children in the working age [24]. With the fading tradition of old-age support, Chinese families are increasingly constrained due to the escalating number of older adults and shrinking number of potential caregivers.

Since 1950 until recent, China’s institutional LTC facilities only admitted those who qualified as “Three Nos” or “Five Guarantees”. The Elderly Care Welfare Institutions (ECWI) was the most common service type. The service receivers were low-income people, such as older adults in absolute poverty, or recipients of public assistance for older adults without family support. The institutional services under LTC began to accept private-payers only from the late 1990s.

In places with little formal capacity to supply LTC, the shift in demographics has further pressured the local governments to develop formal LTC services. Since 2007, the Chinese government has developed ambitious LTC infrastructural projects in partnership with a large number of private enterprises, which marked China’s new era of formal LTC service provision. In the 2010s, the government implemented a series of policies to encourage private enterprises to develop elder care industry. The number of nursing home beds in China has increased annually by an average of 10%, reaching 7.3 million in 2016. China has also achieved the target ratio of 31.6:1000 set in the latest national 5-year plan by 2016, that is, 31.6 beds are now available for every 1000 older adults. During the same time, the number of older adults who received home care has reached 3.2 million by 2016 [25]. The LTC under both nursing home care and home care provides services related to supporting physical and cognitive functions, as well as personal hygiene, to older adults who have difficulty taking care of themselves due to hindrance in daily living activities.

However, in the context of surging demand and supply of LTC services, the coexistence of long waiting lists in some areas and low occupancy rates in others indicates the challenges of equal service distribution [24, 26]. The lack of eligibility criteria in the overarching LTC system makes it difficult to guarantee efficient service delivery to those with urgent LTC needs [27]. Moreover, due to the lack of a holistic process of care provision in conjunction with various underlying care models, the current Chinese LTC system can only offer an acceptable environment with basic standards of living [28]. The rules regulating quality of institutional care and home care only focus on structural aspects of quality, including facilities, built environment, and equipment. There are very few service evaluation studies of LTC for older adults in terms of health outcomes in China, and some results are outdated [29, 30].

Yichang, with a total population of approximately 4 million and located in central China, is the 4th largest city in Hubei Province. According to the Hubei Bureau of Statistics [31], Yichang is one of the most rapidly aging cities in China. By the end of 2015, the number of old people aged 60 and above reached 0.84 million, accounting for 20.8% of the city’s population with local residence [31]. This percentage is higher than the national and provincial average. Although a number of recent research articles have examined older adult care services in metropolitan areas in China (e.g. Beijing, Shanghai, and Guangzhou) [26, 28, 30], limited empirical data are available for small and medium-sized cities. In China, small and medium-sized cities (SMC) play an important role in poverty reduction by improving access to urban services for rural migrants and the poor. Yichang could be a great case to understand long-term care service development in the rise of SMC.

### Pain and long-term care services for older adults: Studies from China and internationally

Older adults, family members, and policymakers are concerned about health outcomes in different care settings, especially nursing home care and home care. However, studies comparing pain prevalence in LTC settings around the world have shown inconsistent findings. For instance, Wysochi et al. [32] conducted a systematic review comparing the health outcomes between home care and institutional care for older adults, and found only two quasi-experiments, which reported no significant difference in pain at baseline or over time between older adults in nursing home care and home care. This review concluded that the quasi-experiments have small sample size and poor quality of methodology, and thus provide low-strength evidence that compare health outcomes between care settings.

There are also few observational studies comparing health outcomes in different LTC settings, and the results are mixed as well. Frytak et al. [33] examined the outcome trajectories of pain in a group of participants from nursing home (*N* = 610) and home care settings (*N* = 605) over a 12-month period. No statistically significant difference in pain was found. Mitchell et al. [34] compared pains of older adults who received care from nursing home (*N* = 3483) and home care settings (*N* = 314) after controlling the baseline participant characteristics. They found that nursing home residents had lower odds with pain compared with home care recipients. Marek et al. [35] compared the pain outcome of older individuals in home care settings (*N* = 78) to individuals of similar case in institutional care (*N* = 78). This study found that pain stabilized in home care recipients while increased in nursing home residents after 6 months. More recently, Blackburn, Locher, and Kilgore [36] conducted a matched retrospective cohort study of Alabamian Medicare beneficiaries (*N* = 12,634), and found that home health beneficiaries averaged 0.2 pain score less than nursing home beneficiaries. This variance in findings across countries is partly due to differences in care settings and study designs, but makes generalizations difficult.

In Chinese population-based studies, much is known about pain characteristics and associated health factors among community-dwelling older adults [37–39]. These studies found that nearly half of the older adults in the studied communities had chronic pain and a negative association was seen between pain and frailty, obesity, and cognitive impairment. Hardly any studies have discussed about pain management services in communities. A number of studies focused on older patients in long-term care facilities [22, 40]. They found that pain was perceived in over 60% of the people in this group and there were limited self-care pain management strategies in nursing homes. But until now, comparative assessment of perceived pain in nursing home and home care settings is missing in the literature.

The review of earlier work clearly demonstrated that the association between LTC settings and pain remains somewhat mixed, and it is still doubtful whether nursing home or home care settings are more positively associated with pain in China. This study compares pain perception in nursing home care and home care among physically dependent older adults in Yichang, China. It departs from earlier research by developing a typology of respondent classification that captures the heterogeneity of Chinese older adults. We attempt to estimate the confounding effect of in a cross-sectional study design with a two-step approach: multivariate regression analysis followed by propensity score matching.

## Methods

### Sampling

The present work is based on an original study conducted in Yichang City in 2015 by the School of Social Development and Public Policy, Beijing Normal University in China. This study was separated into two parts: the first survey targeted the community-dwelling older adults (*N* = 323), and the second focused on nursing home residents (*N* = 402). Both surveys involved older adults aged 65 years and older. The research received the ethical approval from the ethics committee of the School of Social Development & Public Policy at Beijing Normal University in China (reference number: SSDPP-HSC2014003). The sampling followed a multi-stage method as described below.

#### Community-dwelling older adults

We recruited respondents from two districts of Yichang City—Xiling and Wujiagang. These two districts had high concentration of older population. According to the 6th Census, the number of older population in the two districts accounted for over 80% of the older population aged 60 and above in the five urban districts of Yichang. These two selected districts contained 10 sub-districts, six in Xiling and four in Wujiagang. Half of the sub-districts was randomly selected from each district, resulting in five sample sub-districts: Xueyuan, Xiling, and Yemingzhu in Xiling District, and Baotahe and Wanshouqiao in Wujiagang District. Further, we randomly selected our sample from 75 communities among a total of 633 communities in the five selected sub-districts. Here, the ‘community’ refers to one of the smallest political divisions of the People’s Republic of China.

Respondents were recruited through the Municipal Grid Management Center. The number of eligible older adults in the grids varied markedly, ranging from 5 to over 60 and mostly belonged to 30–40 households. Using this as the sampling framework, a total number of 1103 participants were successfully interviewed, which, after checking by the supervisors, produced 1053 valid forms. To be included in this survey, respondents needed to: (1) have a Yichang *hukou* (household registration status); (2) be 65 years or older; and (3) reported needing at least one instrumental activities of daily living (IADL). Therefore, we excluded from the sample 70% of the survey participants who reported no need of any IADL and did not receive any home care services, and ended up with a sample of 323. Trained interviewers at respondents’ homes with written informed consent conducted face-to-face interviews.

#### Nursing home residents

We first obtained a list of all aging care residential facilities in the four urban districts of Yichang with: (1) name; (2) address; (3) year of starting operation; (4) mode of operation (public or private); (5) number of beds; (6) number of residents; and (7) number of residents with different levels of disability assessed by the institutions on their own. This resulted in a total number of 31 residential facilities with 1361 current residents. Then, we chose 22 facilities that had at least 20 residents each for this survey, which covered 97% of the total institutional care residents identified above. Among these, 19 facilities were successfully surveyed. Three facilities declined the survey because of facility renovation.

To select individual participants, we used the random systematic sampling method in each facility. Specifically, in each sampled facility, we first obtained a list of all current residents. Then we used the last digit of the facility director’s mobile phone number and divided it by 3 to reach a remainder A, which became the starting number in selecting the respondents from the name list of all residents in that facility. Face-to-face interviews were conducted by trained interviewers in local nursing homes with older adults’ written informed consent. A total number of 402 residents successfully completed the survey. Response rates in nursing home facilities were greater than 85%.

### Instrument

This study used interRAI Home Care Assessment Form Version 9.1 [41] and interRAI Long-Term Care Facilities Assessment Form and User’s Manual 9.1 [42] as the main data collection and measurement tools for older adults receiving home care and nursing home care. The HC instrument was developed in the mid-1990s, and has been adopted by many developed countries as a standardized tool for assessing old people with disability in home or community settings [36, 43–45]. This instrument was translated and validated in Chinese older population in 2001 [46], and has since been used by a number of social service agencies for assessing LTC needs at the individual level among the Chinese population [47, 48].

The instrument comprises of over 300 clinical and non-clinical items covering 15 domains of functioning: (1) cognition; (2) communication and vision; (3) mood and behavior; (4) psychosocial well-being; (5) functional status; (6) continence; (7) disease diagnoses; (8) health condition; (9) oral and nutritional status; (10) skin condition; (11) medications; (12) treatment and procedures; (13) social supports; (14) environmental assessment; and (15) service utilization. Training material for interviewers was also adjusted for the modified instrument.

### Measurement

Dependent variable: The dependent variable was pain perception. The interRAI scale used two items to create a score from 0 to 2 [49]. This assessment is based on self-reported measures and direct observation by care staff [50]. The scale uses items on pain frequency and pain intensity to create a three-point scale that ranges from no pain (0), mild or moderate pain (1), to daily horrible or excruciating pain (2). This pain scale has been validated in nursing homes and home care settings [49, 51, 52], and used in many studies on Chinese population [2, 5, 39]. Pain was documented if it had occurred in the five days before the assessment in the nursing home group, or within the last three days in the home care group. This difference in observation period in nursing home and home care may slightly reduce the detection of less frequent than daily pain in the interRAI-Home Care but it only affects daily pain that occurs four days ago. The confident interval of this instrument was between 0.617 and 0.759.

Independent variable: The independent variable was residence type of older adults in Yichang City. We classified residence into two mutually exclusive types: nursing home facilities (coded as 1) and home care (coded as 0). These two categories of long-term care settings helped us examine how variations in the type of care facilities have differential effects on pain perception of older adults.

Control variables: Based on the literature review, we found potentially available explanatory factors for pain perception of people with LTC needs. In our analysis, we included individual demographic characteristics (age, gender, education, and marital status), primary caregivers, household size, medical insurance receipt, personal income level, as well as individual health indicators (behavior problems, depression score, mobility, toileting, number of diagnosed chronic diseases, dementia diagnose, presence of pressure ulcer, and inpatient hospital care during the 90-day period prior to the interview).

For individual characteristics, “Level of education” was classified into four categories: no education, primary school, secondary school, and high school and above. Marital status was measured as a three-category variable, not married, currently married, and widowed. We also classified older adults into two mutually exclusive categories based on the local average income level: less than RMB 4000 per month (USD 571, the local average income) or RMB 4000 or above.

For individual health indicators, behavior problems were measured by a score ranging from 0 (no behavior problems) to 2 (frequent behavior problems). Depression was measured by InterRAI Depression Rating Scale, with the range of 0 to 20. Higher score indicated higher probability to be diagnosed as depression case [47]. The Cronbach alpha of this scale was 0.77 based on this sample. The confidence interval was between 2.086 and 2.726. The participants were asked about their mobility, the responses for this question were 0 (Walking with no assistive device), 1 (Walking, uses assistive device), 2 (Wheelchair, scooter), or 3 (Bedbound). Toileting was examined on a 7-point Likert scale (1 = independent, 3 = complete supervision, 5 = complete assistance, 7 = dependent). The participants were asked if they were diagnosed as having any of the following common chronic diseases by doctors, such as hypertension, cervical/lumbar disease, osteoporosis, heart attack, stroke, arthritis, and diabetes. Answers were coded as a binary variable (0 = no, 1 = yes). These scores were then summed to represent the number of diagnosed chronic diseases. Respondents were also asked whether they had been diagnosed as having dementia or Alzheimer’s disease, and answers were classified dichotomously (0 = no, 1 = yes). Presence of pressure ulcer was measured by the number of pressure ulcers on the body. Answers were used to create a binary variable (0 = no, 1 = yes). Finally, the respondents were asked whether they had an inpatient hospital stay in the last 90 days prior to the interview, and the answers were coded as a dichotomous variable (0 = no, 1 = yes).

### Analytical strategy

This study followed a two-step analytical strategy to empirically examine the association between different care modalities and pain perception of older adults. In the first step, we estimated a zero-inflated ordered probit (ZIOP) regression model using residence type as the key independent variable. ZIOP model are used for ordered response variables, when the lower-end zeros that were overly prevalent [53]. The goal is to understand the different effects of home care and nursing home care on the probability of pain perception among older adults, after adjusting for a set of 16 covariates.

In the second step, we performed a stratified propensity score analysis to control for potential selection bias. Propensity score matching method borrows the language of experiments, for instance, treatment group and control group (or treated vs. untreated). In our analysis, the “treatment” and “control” utilized the two long-term care modalities typology in the logistic regression. Specifically, the “control” group is individuals receiving home care, while the “treatment” group is nursing home residents. The logic of propensity score matching method is based on Neyman-Rubin influential counterfactual framework, where propensity score is defined as the conditional probability of receiving treatment giving observed covariates [54]. Once propensity scores have been estimated, and the treated and control groups have similar propensity scores, the observed covariates are automatically controlled. Therefore, any differences between the treatment and control groups would be attributed to the receipt of treatment, and this is not as a result of observed covariates. This adjustment accomplishes improved control for confounding [55].

For the purpose of this analysis, we used *psmatch2*, a user developed program available in Stata 14.0, which contains *pscore* and *att* programs [56]. In addition, we also conducted paired *t*-test with the propensity-score-matched groups to compare the difference in pain perception between the two matched groups. All reported *P* values are 2-sided, and *P* values less than 0.05 were considered to indicate statistical significance.

In addition, the percentage of missingness of all variables was lower than 5% (*N* = 5). Listwise deletion was used to handle missing data, resulting in a final sample size of 720 (400 from nursing homes and 320 from communities).

Sample size was calculated using the Power and Sample Size Calculation software (version 3.1.2, 2014) [57]). The appropriate sample size for detecting a difference between two proportions (60% vs. 40%) was 95 at 80% power and 95% confidence level. With 720 observations, this sample has achieved appropriate size for detecting a difference between home care and nursing home care recipients.

## Results

### Descriptive statistics

Descriptive statistics are presented in Table 1 to summarize characteristics of the sample and examine distributions of dependent and independent variables as well as the covariates. A total of 720 participants met the study inclusion criteria. Overall, 55.6% of the sample was composed of nursing home residents and 45.4% were community-dwelling older adults. The sample included more women respondents (61.1%) than men (38.9%). Nearly 59.7% of the older adults were widowed, 5.6% were not married, and 34.7% were married with spouse present. The vast majority of older adults (93.5%) were covered by health insurance. Most of the older adults had spouse as the primary caregiver (53.1%), nearly 36.5% reported children as the primary caregiver, and 10.4% had no family caregiver. The education levels of the older adults varied: 26.9% had no education, 31% had primary education, 34.9% had secondary education, and only 7.2% received high school and above education.

**Table 1:** Descriptive Characteristics of LTC Study Participants in Yichang China, According to Residence, Before and After Propensity-Score Matching

			Unmatched					Matched				
	Total		Home		Nursing Home			Home		Nursing Home		
	Mean(%)	SD	Mean(%)	SD	Mean(%)	SD	p value	Mean(%)	SD	Mean(%)	SD	p value
Pain												
0. No pain	55.03%		62.64%		49.00%			54.60%		48.56%		
1. Mild/moderate pain	23.86%		17.65%		28.86%			31.50%		29.92%		
2. Daily horrible/excruciating pain	21.10%		19.81%		22.14%			13.91%		21.52%		
Residence												
Home	45.44%											
Nursing home	55.56%											
Age	77.80	7.76	77.32	7.11	78.20	8.03	0.014	79.55	7.15	78.20	8.03	0.129
Gender												
Female	61.11%		72.70%		60.60%		<.0001	62.00%		60.60%		0.712
Male	38.89%		27.30%		39.40%			38.00%		39.40%		
Marital status												
Not married	5.56%		2.80%		7.61%		<.0001	10.24%		7.61%		0.204
Married with spouse present	34.72%		53.58%		19.42%			15.75%		19.42%		
Widowed	59.72%		43.61%		72.97%			74.02%		72.97%		
Education												
No education	26.94%		24.61%		29.40%		0.014	35.70%		29.40%		0.156
Primary	30.97%		37.07%		24.93%			30.71%		24.93%		
Secondary	34.86%		28.87%		38.06%			31.15%		38.06%		
High school and above	7.22%		4.72%		7.61%			7.17%		7.61%		
Primary caregiver												
None	10.40%		8.72%		12.07%		<.0001	11.82%		12.07%		0.15
Spouse	53.12%		46.42%		58.79%			57.88%		58.79%		
Children	36.48%		44.86%		29.13%			30.30%		29.13%		
Have insurance	93.47%		97.51%		89.76%		<.0001	88.22%		89.76%		0.822
Income more than 4000 RMB	59.95%		44.58%		71.20%		< 0.001	54.26%		70.34%		0.31
Mean of behavior problems (range:0–2)	0.04	0.16	0.02	0.12	0.04	0.17	<.0001	0.04	0.15	0.04	0.17	0.745
Depression score (range:0–20)	1.97	3.29	1.08	4.65	2.55	3.59	0.004	2.38	2.41	2.55	3.59	0.601
Mobility												
Independent	66.11%		78.51%		58.79%		<.0001	58.81%		58.79%		0.81
Walks with assistance	22.92%		17.45%		28.87%			28.08%		28.87%		
Wheelchair-dependent	8.33%		4.05%		12.34%			13.12%		12.34%		
Bedbound	2.64%											
Toileting												
Independent	79.17%		80.06%		78.25%		0.002	79.06%		78.25%		0.481
Some supervision	5.14%		5.92%		4.46%			4.20%		4.46%		
complete supervision	0.83%		2.25%		2.53%			2.46%		2.53%		
some assistance	2.92%		2.80%		3.15%			3.20%		3.15%		
complete assistance	3.19%		3.43%		3.63%			2.73%		3.63%		
Most assistance	0.83%		0.62%		0.79%			0.62%		0.79%		
Dependent	7.92%		1.89%		6.30%			6.22%		6.30%		
Dementia diagnose	10.99%		9.29%		12.24%		<.0001	9.69%		10.15%		0.942
No. of chronic disease (range:0–20)	0.22	0.16	0.26		0.19			0.20		0.19		0.673
Have at least one ulcer	2.50%		1.05%		2.63%		0.016	2.28%		2.63%		0.704
Have inpatient in the last 90 days	16.11%		22.57%		13.65%		0.013	15.32%		13.65%		0.403
Observations	720		320		400			242		242		

*SD* standard deviation

With regard to pain perception, about 55% of the respondents reported no pain. The percentage of nursing home residents who reported daily horrible pain (22%) was higher than those in home care (20%). The majority (66.1%) of the respondents was independent in terms of mobility, 22.9% reported walking with assistance, 8.3% were wheelchair-dependent, and 2.6% were bedbound. On average, the number of behavior problems was 0.04, with a depression score of 1.97. Most of the respondents reported toileting independence (79.2%), nearly 21% of the older adults needed assistance with toileting. Approximately 16% of the sample had an inpatient hospital stay in the last 90 days. The average number of chronic conditions is 0.22. Less than 10% of the respondents were diagnosed with dementia. A small proportion of the older adults had one or more ulcers (2.5%) (Table 1).

The characteristics of participants who received home care were compared with the nursing home care recipients in Table 1. The likelihood of receiving nursing home care was significantly greater for older adults who were of older age, female, widowed, and less educated, had spouse as primary caregiver, and had less medical insurance coverage compared with those receiving home care. A greater proportion of nursing home care recipients demonstrated worse mobility and toileting independence and had a higher presence of pressure ulcers. On average, nursing home care recipients had more diagnosed chronic conditions and dementia. Behavioral problems and depression were worse in nursing home care recipients. However, a notable 22.6% home care recipients had an inpatient hospital stay in the last 90 days, more than nursing home care recipients.

In order to control for the differences in confounding variables, propensity score matching was conducted. After propensity score matching was completed, there were 242 matched pairs of respondents. Unlike the substantial differences between the unmatched respondents in these two groups, there were no significant differences between the home care group and the nursing home care group in any of the 14 covariates. Table 1 also presents the estimated absolute standardized differences for all covariates to assess the pre-match imbalances and post-match balances. *p-*values larger than 0.05 suggested no bias, and *p*-values less than 0.001 indicated inconsequential bias.

Table 2 describes the treatment and control sample sizes for each propensity score stratum. For instance, in the first propensity score stratum [0.00, 0.25], the control group (community dwelling) had a sample size of 115 and the associated treatment group (nursing home care) had a sample size of 16.

**Table 2:** “Treatment” and “Control” Sample Sizes per Propensity Score Stratum (*N* = 720)

Propensity score strata	Residence	
	N (control) *community	N(treatment) *nursing home
(0.00–0.25)	115	16
(0.25–0.50)	106	90
(0.50–0.75)	75	125
(0.75–1)	24	169
*N*	320	400

### Multivariate results

Table 3 presents estimates of the causal effect for pain of older adults receiving nursing home care. The estimates were obtained from ZIOP regressions and propensity score adjusted regressions. Each estimate can be interpreted as the adjusted difference in mean outcomes for nursing home residents minus the mean outcomes for the community-dwelling home care recipients so that the size of the estimate stands for the average treatment effect of the treated (ATT). Standard errors and sample sizes were provided for each comparison.

**Table 3:** Zero-inflated ordered probit regression estimates of pain Among Older Adults in Yichang, China

	Zero-inflated ordered probit regression (unmatched)			Propensity score adjusted regression (matched)		
	Coef.	Std. Err.	P > z	Coef.	Std. Err.	P > z
Nursing home	0.088	0.196	0.655	0.489	0.169	0.004
Age	0.002	0.012	0.833	0.000	0.010	0.992
Male	−0.403	0.165	0.015	−0.538	0.138	0.000
Marital status						
(ref. not married)						
Married with spouse present	−0.826	0.418	0.048	0.227	0.286	0.428
Widowed	−0.634	0.433	0.143	0.038	0.275	0.891
Education attainment						
(ref. no education)						
Primary school	−0.489	0.235	0.038	0.001	0.176	0.997
Secondary school	−0.186	0.218	0.393	−0.110	0.173	0.524
High school and above	−0.569	0.302	0.060	−0.393	0.264	0.137
Primary caregiver						
(ref. None)						
Spouse	0.836	0.308	0.007	0.086	0.207	0.677
Children	0.831	0.305	0.006	0.119	0.215	0.580
Have insurance	−0.105	0.321	0.744	−0.181	0.234	0.438
Monthly income						
(ref. less than 4000 RMB)						
4000+ RMB	−0.310	0.186	0.096	−0.087	0.145	0.549
No. of behavioral problems	−0.941	0.467	0.044	−0.314	0.369	0.394
Depression score	0.066	0.024	0.007	0.042	0.020	0.035
Mobility						
(ref. independent)						
Walks with assistance	−0.185	0.184	0.316	0.073	0.153	0.631
Wheelchair-dependent	0.782	0.315	0.013	−0.067	0.240	0.779
Bedbound	–	–		–	–	
Toileting						
(ref. Independent)						
Some supervision	0.334	0.356	0.348	0.140	0.303	0.644
Complete supervision	1.201	0.656	0.067	0.568	1.118	0.611
Some assistance	0.674	0.585	0.249	0.150	0.419	0.720
Complete assistance	0.321	0.504	0.524	0.197	0.424	0.642
Most assistance	1.251	0.634	0.049	0.353	0.736	0.631
Dependent	0.195	0.370	0.598	0.207	0.290	0.475
Have dementia	0.034	0.265	0.897	0.023	0.217	0.917
No. of chronic disease	1.231	0.579	0.033	2.438	0.520	0.000
Have at least one ulcer	1.791	0.808	0.027	0.864	0.471	0.067
Had inpatient service in last 90 days	0.288	0.220	0.190	0.098	0.180	0.586
Constant	−0.088	0.928	0.924	−0.713	0.807	0.377
cut1	−0.864	1.881		−2.008	9.759	
cut2	3.240	1.760		2.589	1.234	
AIC	1246.717			941.125		
BIC	1471.536			1165.944		
N	484			484		

For approximating the power of the ordered probit model, a zero-inflated ordered regression of pain (range: 0–2) on a dichotomous variable (residence) with a sample size of 720 observations achieves 86% power at a 0.05 significance level to detect a change in Prob(Y = 1) from the value of 0.58 at the mean of X to 0.52 when X is increased to one standard deviation above the mean. This change corresponds to an odds ratio of 0.78. An adjustment was made since a multiple regression of the independent variable of interest on the other independent variables in the ordered probit regression obtained an R-Squared of 0.1.

#### Results of unadjusted ZIOP regression model

Moving across Table 3, the estimates from ZIOP regressions with complete cases (*N* = 484) in the second column show nursing home care were not statistically significantly predictive of pain. That is, as compared with community-dwelling home care recipients, nursing home residents were not more likely to perceive pain (*β* = 0.088, SE = 0.196, *p* = 0.142). The average marginal effects were insignificant (dy/dx = − 0.026, SE = 0.059, *p* = 0.658). These results indicate that the difference between the two groups was insignificant but clinically relevant. We conducted the Vuong test and confirmed that the inflation part of the model is necessary (*z* = 4.43, Pr > *z* = 0.00) [58, 59]. Compared with classical likelihood-ratio test, this ZIOP model has smaller AIC and BIC values, indicating that it fits the data better [60].

#### Results of propensity score adjusted regression model

When using non-experimental design data, a limitation of multivariate analysis is that the approach does not address the problem of selection bias derived from embedded difference between the treatment and control groups. In this study, we found heterogeneities in nursing home care choices by marital status and primary caregiver. We addressed the heterogeneities by using propensity score adjusted regression, balancing the treatment and control groups, and ensuring the two groups are statistically similar at baseline. Overall, after controlling the confounding factors, results from propensity score adjusted regression are similar with those of multivariate ZIOP regression.

The estimates of propensity score adjusted regression suggest that older adults in nursing home care are more likely to perceive pain compared with those in home care settings (*β* = 0.489, SE = 0.169, *p* = 0.004). We also calculated predictions of the probability of nonparticipation (average marginal effects), which in this example means the probability of perceiving no pain (dy/dx = − 0.169, SE = 0.056, *p* = 0.03) [59]. The estimates of propensity score adjusted regression revealed that all covariates being equal, on average in the data, the home care group is about 16.9% less likely to perceive pain than nursing home residents. The coefficient became significant, and magnitude of the coefficient in the propensity score matching regression model was larger than ZIOP regression estimates. This finding indicates that living in nursing home would increase the likelihood of pain perception. Consistent with studies conducted on China, pain is important to determine nursing home care choices, suggesting the crucial role of pain management in older adults’ long-term care needs.

#### Estimates of covariates

It is worthwhile to point out that some covariates were statistically significantly associated with pain. Results from ZIOP regression estimates in the second column of Table 3 suggest that 10 covariates [i.e., male (*β* = − 0.403, SE = 0.165, *p =* 0.015), primary school (*β* = − 0.489, SE = 0.235, *p =* 0.038), Spouse as primary caregiver (*β* = − 0.836, SE = 0.308, *p =* 0.007), children as primary caregiver (*β* = 0.831, SE = 0.305, *p* = 0.006), number of behavioral problems (*β* = − 0.941, SE = 0.467, *p* = 0.044), depression score (*β* = 0.066, SE = 0.024, *p* = 0.007), wheelchair-dependent (*β* = 0.782, SE = 0.315, *p* = 0.013), needs most assistance for toileting (*β* = 1.251, SE = 0.634, *p* = 0.049), number of chronic diseases (*β* = 1.231, SE = 0.579, *p* = 0.033), and have at least one ulcer (*β* = 1.791, SE = 0.808, *p* = 0.027)] were statistically significantly associated with experiencing pain.

Compared with the results from the conventional multivariate ZIOP regression, the estimates of propensity score adjusted regression (column 3, Table 3) suggest fewer covariates that were statistically significantly associated with pain. Three covariates were statistically significantly associated with the propensity to experiencing pain: male (*β* = − 0.538, SE = 0.138, *p* = 0.000), depression score (*β* = 0.042, SE = 0.020, *p* = 0.035), number of chronic disease (*β* = 2.438, SE = 0.520, *p* = 0.000). Interestingly, in propensity score adjusted regression the coefficient of depression score decreased, while the coefficient of male and number of chronic disease increased compared with ZIOP regression estimates.

## Discussion

Using the interRAI local survey data from Yichang City, China, this study investigated whether living in nursing home or at home would pain perception of older adults. The research models apply both ZIOP regression analysis and propensity score matching method while adjusted for participant characteristics. The results affirm that pain perception of Chinese older adults in nursing home facilities was significantly more severe than that of older adults receiving home care. This finding is in line with the results of Blackburn, Locher, and Kilgore [36] and Marek et al. [35] who reported less pain among older adults in home care settings than those nursing home residents. However, the results of this study are not consistent with other studies that found no significant difference in pain perception between nursing home residents and home care recipients [33].

There are at least two potential explanations for the finding that home care recipients had less pain than older adults in nursing home. The first explanation may be the inadequate staffing of nursing homes with nurses and care workers. Kane et al. [19], Harrington [61], and Jette, Warren, and Wirtalla [62] reported that the majority of nursing homes in the United States failed to prepare adequate staff to provide pain management. Lower nursing staff levels and pain management intensity are related to worsen pain prevalence. Similarly, the staffing levels of nurses and care workers are also inadequate in the majority of nursing homes in China. Older adults tend to choose public nursing home because of medical insurance and low expenses. According to the current Chinese national standards, public nursing home can only guarantee older adults an acceptable living space with minimal reasonable standards of living. Regulations regarding personnel require minimum one physician and two registered nurses present in one facility, and one nursing assistant per 10 patients [63]. However, to reduce cost, nursing assistants are also in charge of cleaning and preparing meals in daily tasks. The inadequate staffing of nurses and care workers leads to deficiencies in health care services that may exacerbate pain perception of older adults in nursing homes. It is impossible to relieve the pain without sufficient and high-quality health care services.

Another possible explanation is the important contribution of family care. As many studies have noted, pain is probably best understood with a biopsychosocial approach, which takes into account not only pathology but also individual, social, and cultural differences that may affect perception of and response to pain [10, 18]. At home, older adults are more likely to feel secure and relaxed with family members compared with those in nursing homes. The present results are consistent with Chou and Chi [5] who found older adults with informal caregivers felt less depressed and pain than those without informal caregivers. Our results may also reflect Chinese cultural norms related to the expression of feelings of pain: older adults who receive home care tend to underreport pain perception in order to reduce family caregiver’s emotional burden.

Moreover, this study surprisingly shows that 78.51% of nursing home residents reported independence in toileting and 58.79% in mobility. This result is in contrast to most current studies from developed countries that report frail older adults are apt to stay in nursing home rather than at home. The possible explanation for this is the lack of assessment system for admission in nursing homes when the Chinese government began long-term care expansion in the 2010s. During the recent expansion of nursing homes in China, allocating residential care resources relied only on the ability of the care recipients or family to pay rather than was based on needs.

These findings point to a crucial demand to develop home care services for older adults. Developed countries have attempted de-institutionalizing long-term care system [64], which encourages the shift of long-term care modality from institutional care settings to community-based care services due to cost and consumer preference [65]. However, in China, professional home care services have not been well-developed. Currently, home care only provides assistance without case management and professional nursing services. Despite these conditions, this research indicates that home care services could relieve pain at less expense than institutional care. In China, nursing home residents are eligible for reimbursement of cost equivalent to USD 71 per month based on a national standard [53]. Meanwhile, older adults receiving qualified home care services are eligible for a lower benefit of maximum USD 29 per month [66]. In the future, government should invest and develop higher quality home care and make services available in order to meet the increasing demand of Chinese older adults.

The results of this study also suggest that it is necessary to develop clinical strategies for pain management in nursing homes. The staffing levels of registered nurses and social workers should enable adequate health services and emotional support in nursing homes in order to relieve the pain of older adults. In-service training about pain recognition and treatment is recommended for nursing home health providers in China. Introduction of pain assessments at regular times, treatment based on pain guidelines, and interdisciplinary discussion of assessment and treatment may help to achieve this goal. An improvement in the caregivers’ knowledge of pain assessment and management is also necessary in the care of older adults.

We note some limitations of this study. First, the results may not be generalizable to all older urban Chinese adults given the unique societal and cultural factors at play in Yichang City. Second, the interRAI pain items might be insensitive in detecting pain among older adults with cognitive impairment and dementia [21]. This could result in an underestimate of the strength of the association between dementia diagnose and pain prevalence. Our findings show no association between accessed pain and dementia diagnoses, which reflect the limited clinical utility of the interRAI pain items. This tool is also limited in providing information on the type of pain experienced and its cause. Third, the pain was assessed by interviewing residents and direct observation by care staff. Self-assessment of diagnoses is likely of low validity and the potential for underestimation remains a concern. This might be a possible factor that may influence the results, especially in older adults suffering from dementia.

Fourth, although we confirm the empirical validity of a positive association between nursing home care and pain among older adults, we have not yet addressed the pathways through which this happens. The statistical models have been able to rule out possibilities of confounding (e.g. age, health insurance, dementia diagnose, family caregivers), but we have not delineated the pathways. Pain perception in different care modalities is a complex and subjective process, and a careful investigation of these mechanisms remained outside the scope of the present study. Although we observed different pain prevalence patterns between home care and nursing homes, we did not assess the motivating factors, which might be rooted in staffing levels, therapy intensity, and family support. We believe these remain important themes for future efforts in long-term care research in China.

We have earlier argued that this study has offered stronger empirical support for the association between LTC services and pain perception in China. This is not to suggest that we have established causality. Rather, we would like to note that even though propensity score matching balance observed covariates between the control and treatment groups and reduce the confounding effects, this method still cannot balance unmeasured characteristics. Therefore, interpretation of this study can be better described as supporting an association rather than causation. The variables used in this study were limited to the LTC dataset in Yichang City, and potentially important variables, such as detailed information on services and interventions, were not available. We expect that the high-quality panel data collected through future surveys could provide a useful next step to examine these causal mechanisms more appropriately. These limitations notwithstanding, evidence from this study highlights the importance of developing LTC services delivered at an individual’s home. Identifying crucial pathways will considerably advance future research in the realm of long-term care research in China.

## Conclusions

This study finds that older adults in nursing home showed more severe pain than those who received home care after controlling for participants’ characteristics. These findings suggest that professional home care services and support programs encouraging older adults aging-in-place might be a desirable policy strategy to help relieve pain perception of older adults who otherwise would not be able live independently and need to move to institutional care settings.

## Abbreviations

ADL: Activities of daily living
ATT: Average treatment effect of the treated
ECWI: Elderly care welfare institutions
IADL: Instrumental activities of daily living
LTC: Long-term care
SMC: Small and medium-sized cities
ZIOP: Zero-inflation ordered probit
